# Usefulness of laparoscopy in the management of incidentally discovered retroperitoneal liposarcoma mimicking inguinal hernia: A case report and literature review

**DOI:** 10.1016/j.ijscr.2020.05.064

**Published:** 2020-06-03

**Authors:** Takashi Matsumoto, Kosuke Mima, Asuka Ono, Nobutomo Miyanari, Atsushi Morito, Shinsei Yumoto, Keisuke Kosumi, Mitsuhiro Inoue, Takao Mizumto, Tatsuo Kubota, Hideo Baba

**Affiliations:** a Department of Surgery, National Hospital Organization Kumamoto Medical Center, 1-5 Ninomaru, Chuo-ku, Kumamoto, 860-008, Japan; b Department of Gastroenterological Surgery, Graduate School of Medical Sciences, Kumamoto University, 1-1-1 Honjo, Chuo-ku, Kumamoto, 860-8556, Japan

**Keywords:** CT, computed tomography, MRI, magnetic resonance imaging, Sarcoma, Retroperitoneum, Transabdominal preperitoneal repair

## Abstract

**Introduction:**

Liposarcomas comprise around 10%–16% of soft tissue sarcomas. The two major sites of liposarcoma are the extremities and retroperitoneum. However, retroperitoneal liposarcomas mimicking inguinal hernia are rare. We present a case of retroperitoneal liposarcoma mimicking inguinal hernia, which was diagnosed after laparoscopic surgery and underwent curative resection.

**Presentation of case:**

A 46-year-old man was admitted to our hospital with a right inguinal pain and swelling that had been recognized for 3 years. We diagnosed the inguinal swelling as a right inguinal hernia and planned laparoscopic surgery for inguinal hernia repair. A hernia sac, however, was not found and swollen retroperitoneal fatty tissue near the right internal inguinal ring was observed by laparoscopy. We aborted the surgical procedure and performed computed tomography and magnetic resonance imaging, which revealed an extraperitoneal and lipomatous tumor extending through the inguinal canal to the scrotum. Wide local excision of the tumor, along with right orchidectomy, was performed under laparotomy. Histopathological diagnosis showed well-differentiated liposarcoma of the retroperitoneum and confirmed tumor-free margins. No evidence of recurrence or metastasis was seen in the 9 months after curative resection.

**Discussion and conclusion:**

Laparoscopic surgery for inguinal hernia enables to observation of the inguinal region and management of rare cases, such as retroperitoneal liposarcoma.

## Highlights

• We present a rare case of retroperitoneal liposarcoma with an inguinal swelling, which was diagnosed after laparoscopic surgery for inguinal hernia.
• It is necessary to consider a possibility of enlargement of retroperitoneal liposarcoma and perform CT or MRI of the abdomen and pelvis in cases of irreducible inguinal swelling of retroperitoneal fatty tissue.

## 1 Introduction

Soft tissue sarcomas are rare tumors that represent 1% of all diagnosed malignant neoplasms, and liposarcomas account for 9.8%–16% of soft tissue sarcomas [1,2]. The two major sites of liposarcoma are the extremities and retroperitoneum [3]. However, inguinal liposarcomas are rare. We present a rare case of retroperitoneal liposarcoma with an inguinal swelling, which was diagnosed after laparoscopic surgery for inguinal hernia. This work has been reported in line with the SCARE criteria [4].

## 2 Presentation of case

**Fig. 1:**
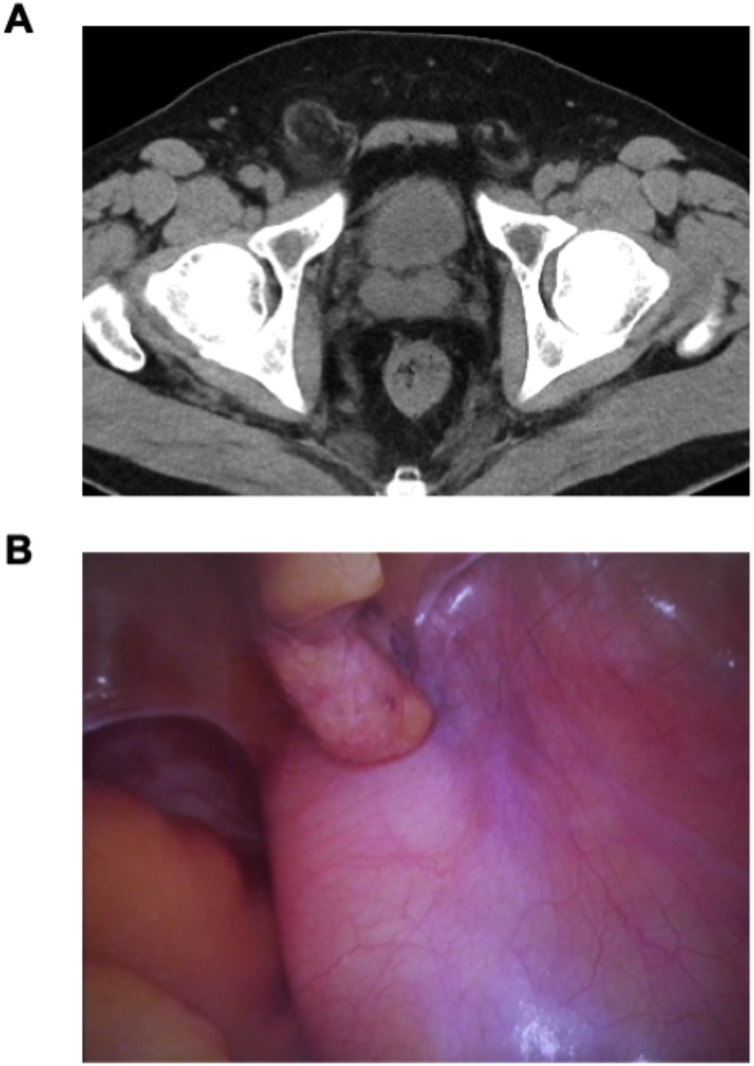
(A) Non-contrast computed tomography suggested an inguinal herniation. (B) Laparoscopic image of swollen retroperitoneal soft tissues.

**Fig. 2:**
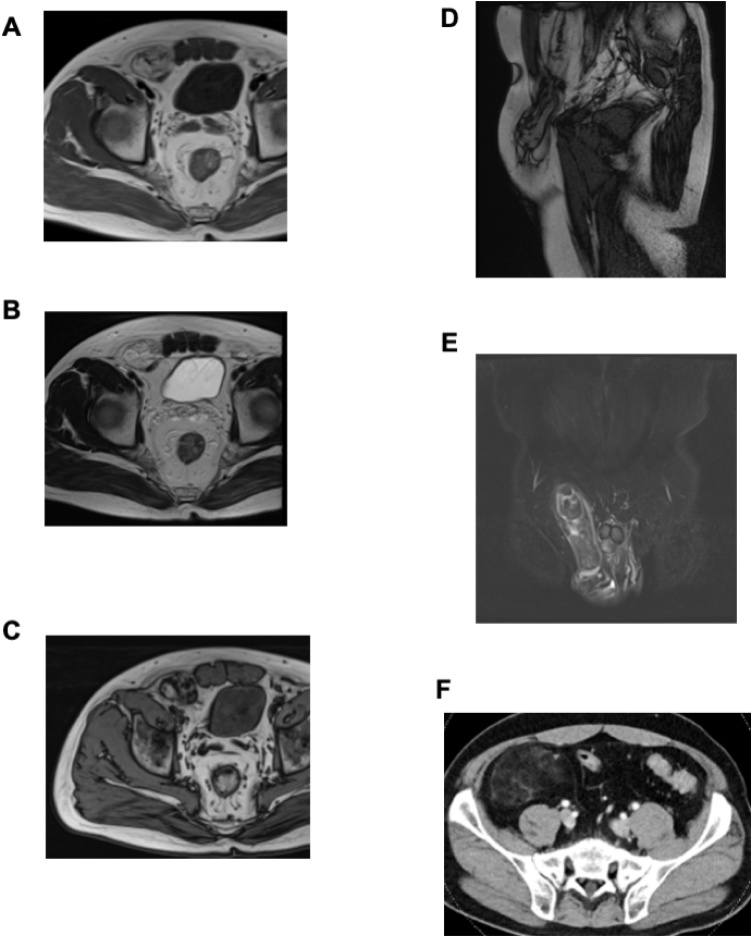
(A) Contrast-enhanced magnetic resonance imaging, showing partial solid components with low in T1 emphasized. (B) High in T2 emphasized. (C) Fatty components with high both in T1 and in T2 emphasized, however low in fat suppression images. (D) Sagittal image in T2. (E) Coronal image in T2 STIR. (F) Contrast-enhanced computed tomography revealed a tumor, including fatty tissue, measuring 80 × 60 mm in front of the major psoas muscle in the hypogastric region.

A 46-year-old man presented with a right inguinal swelling 3 years ago and inguinal pain was gradually strong over half a year, but he had no digestive symptoms like a nausea, vomit, and constipation. He was diagnosed with right inguinal hernia and admitted to our hospital. His blood test findings were normal. Physical examination showed an irreducible swelling in the right inguinal region in a standing position, and the other side was normal. The right inguinal swelling did not stand out in a supine position. The inguinal bulge was soft and had no pulse. In our group, we perform non-contrast computed tomography (CT) before the operation routinely. We check the inguinal area, contents and status of the other side. At the first consultation, an adipose tissue like a greater omentum entered in a right inguinal area in non-contrast CT before surgery (Fig. 1A). In addition, he complained of inguinal pain recently. We diagnosed a right inguinal hernia. We intended to use a laparoscopic transabdominal approach for inguinal hernia repair. A hernia sac, however, was not found and swollen retroperitoneal fatty tissue near the right internal inguinal ring was observed by laparoscopy (Fig. 1B). The fatty tissue was not invasive to the peritoneum and we could not verify the collect margin. Because we considered the risk of tumor cell seeding following biopsy, we did not perform a tumor biopsy. We aborted the surgical procedure and performed contrast-enhanced CT for evaluating vascular invasion, distant metastasis, and positional relationship between tumor and blood vessels in more detail. Magnetic resonance imaging (MRI) was performed for evaluating properties of the soft tissue in detail. Findings of contrast-enhanced CT and MRI revealed an extraperitoneal and lipomatous tumor measuring 80 × 60 mm, extending through the inguinal canal to the scrotum (Fig. 2). And then, we decided the margin of the resection before second surgery on the basis of the CT and MRI findings. Because of the risk of tumor cell seeding following biopsy and suspicion for tumor invasion of right spermatic cord or scrotum, we aimed for primary complete curative resection along with right orchiectomy to achieve negative margins without any pathological samples. By means of several information and findings about the inguinal mass, we explained the possibility of liposarcoma and extended resection for right orchiectomy and got patient’s consent in the informed consent. We performed wide local excision of the tumor, along with right orchidectomy. The patient’s postoperative course was unremarkable, and he was discharged on postoperative day 13.

**Fig. 3:**
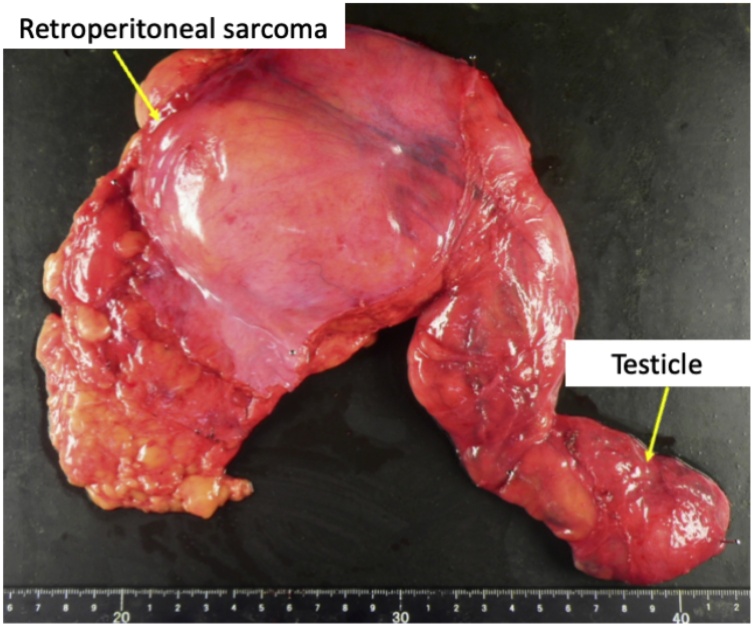
Resected tumor measuring 280 × 200 × 180 mm.

**Fig. 4:**
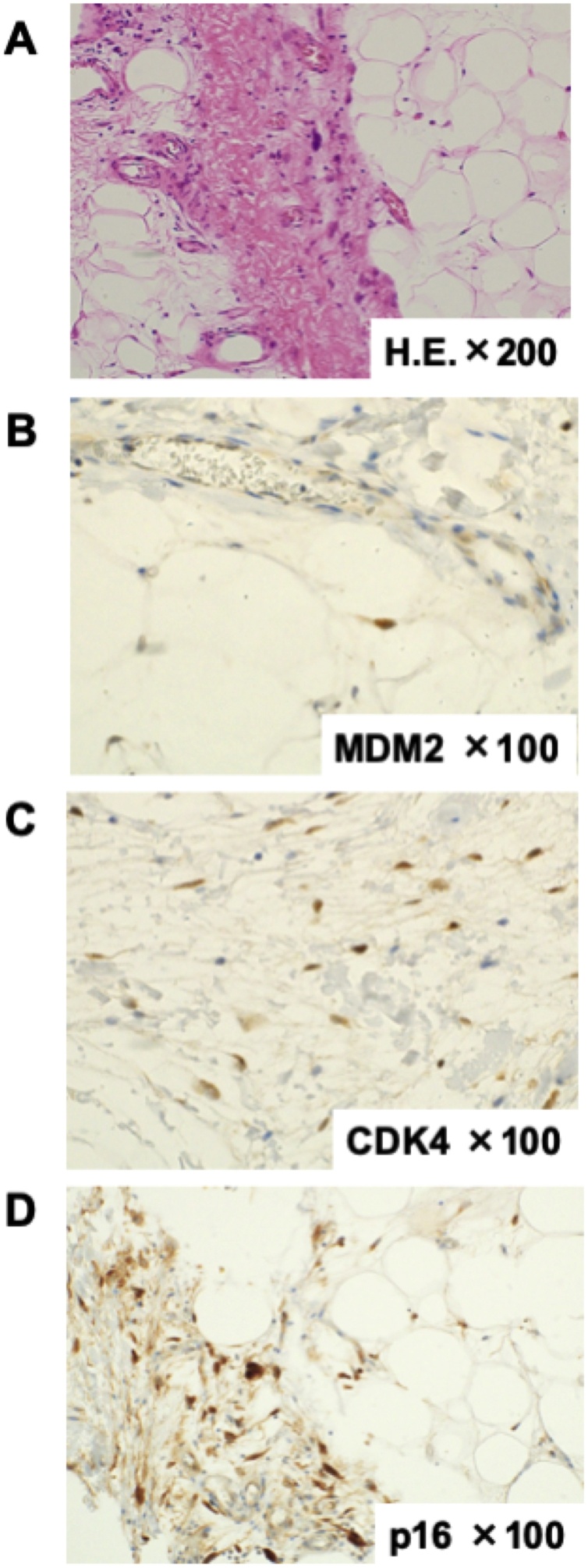
(A) Hematoxylin-eosin staining (200× magnification). (B) mouse double minute 2 immunostaining (100× magnification). (C) Cyclin-dependent kinase-4 immunostaining (100× magnification). (D) p16 immunostaining (100× magnification).

The resected tumor measured 28 × 20 × 18 cm and weighed 510 g (Fig. 3). Positive immunostaining for mouse double minute 2, cyclin dependent kinase-4, and p16 confirmed histopathological diagnosis of a well-differentiated retroperitoneal liposarcoma (Fig. 4). No malignant cells were found in any of the surgical margins. Periodic follow-up was performed and no evidence of recurrence or metastasis was seen in the 9 months after curative resection.

## 3 Discussion

Liposarcomas account for 9.8%–16% of soft tissue sarcomas [1,2]. Retroperitoneal liposarcoma is primarily a tumor of adults with a peak incidence between 50 and 65 years [10]. Bhandarwar et al. reported that retroperitoneal liposarcoma has three principle forms: (1) atypical lipomatous tumor well-differentiated (50%–55%); (2) myxoid-round cell variety (40%); and (3) pleomorphic (5%) [10]. Liposarcomas frequently originate in the extremities or retroperitoneum [16], but inguinal liposarcomas are rare. The retroperitoneal cavity communicates with the inguinal region through cord structures, and hence retroperitoneal liposarcoma can occasionally extend through the inguinal canal into the scrotal sac, presenting as an indirect inguinal hernia. Furthermore, retroperitoneal liposarcoma grows slowly and presents non-specific symptoms, except for irreducible inguinal swelling [5].

A review of literature showed 12 cases of retroperitoneal liposarcoma initially presenting as inguinal hernia, including our present case (Table 1). The age of the patients at presentation ranged from 40 to 86 years with an average of 59.3 years. The size of the tumor at presentation also varied, ranging from 17 to 48 cm with an average of 32.9 cm. Six of the 12 reported cases were diagnosed preoperatively as inguinal hernia. In postoperative histopathological specimens, 10 patients showed well-differentiated liposarcomas and two were pleomorphic.

**Table 1:** Literature review of cases of retroperitoneal liposarcoma diagnosed by inguinal swelling.

	Age/sex	Size (cm)	Right/Left	Type	Initial diagnosis
Noguchi et al. (2001) [6]	60/M	28	Right	Pleomorphic	Hernia
Mizuno et al. (2006) [7]	53/M	45	Left	Well-differentiated	Tumor
Baldassarre et al. (2007) [8]	69/M	NA	Left	Well-differentiated	Hernia
Ghimire et al. (2011) [9]	53/M	28	Right	Well-differentiated	Tumor
Bhandarwar et al. (2011) [10]	40/M	47	Right	Well-differentiated	Tumor
Leão et al. (2012) [11]	86/M	30	Right	Well-differentiated	Hernia
McKinley et al. (2013) [12]	63/M	17	Right	Well-differentiated	Tumor
Tardu et al. (2016) [13]	53/M	48	Left	Pleomorphic	Hernia
Fiaschetti et al. (2017) [14]	64/M	21	Left	Well-differentiated	Tumor
Fiaschetti et al. (2017) [14]	67/M	24	Left	Well-differentiated	Tumor
Lechner et al. (2019) [15]	57/M	46	Right	Well-differentiated	Hernia
Present case	47/M	28	Right	Well-differentiated	Hernia

M, male; NA, not available.

Inguinal hernia surgery is performed in many patients worldwide. The types of operation vary and include the anterior approach and laparoscopy [17]. Table 2 summarizes the approaches and outcomes of surgery for retroperitoneal liposarcoma diagnosed as inguinal hernia. Anterior approaches for hernia could not remove the tumors completely in any patients, and reoperation was required for the residual tumors. In those cases, it was difficult to diagnose the liposarcoma intraoperatively because in the inguinal region the tumor was not invasive and appeared homogeneous and light-yellow in color, similar to normal fat or lipoma. In contrast, in the present case and one other, a laparoscopic transabdominal approach for inguinal hernia repair was intended. Retroperitoneal tumors were detected during laparoscopy, and curatively resected after CT and MRI. Laparoscopic surgery for inguinal hernia repair may be able to observe the inguinal region and manage rare cases, such as retroperitoneal liposarcoma.

**Table 2:** Surgical approaches and outcomes for six retroperitoneal liposarcomas diagnosed as inguinal hernias.

Source	Age/sex	Size (cm)	Approach for hernia	Outcome
Noguchi et al. (2001) [6]	60/M	24	Anterior Open	Reoperation for residual tumor
Baldassarre et al. (2007) [8]	69/M	NA	Anterior Open	Reoperation for residual tumor
Leão et al. (2012) [11]	86/M	30	Anterior Open	Reoperation for residual tumor
Tardu et al. (2016) [13]	53/M	48	Anterior Open	Reoperation for residual tumor
Lechner et al. (2019) [15]	57/M	46	Laparoscopy	Imaging studies and curative resection
Present case	47/M	28	Laparoscopy	Imaging studies and curative resection

Curative surgical resection of the tumor is the most effective treatment of liposarcoma. In the present case, we performed wide local excision of the tumor along with right orchidectomy, and achieved curative resection. A previous report suggests that orchidectomy via the inguinal approach should be performed for paratesticular liposarcoma [18]. Histopathological diagnosis showed well-differentiated retroperitoneal liposarcoma. Periodic follow-up was performed and there was no evidence of recurrence or metastasis in the 9 months after the second operation. No adjuvant therapy was performed. According to various reports, liposarcomas have a high incidence of recurrence of 21%–83% [16,19]. Enterline et al. reported that 31% of all patients with liposarcoma developed distant metastasis [20]. In contrast, well-differentiated liposarcomas have intrinsically low-grade malignancy and recur but do not metastasize [19]. Therefore, in this case, careful follow-up was necessary to ensure that there was no local recurrence.

## 4 Conclusion

It is necessary to consider a possibility of enlargement of retroperitoneal liposarcoma and perform CT or MRI of the abdomen and pelvis in cases of irreducible inguinal swelling of retroperitoneal fatty tissue. Laparoscopic surgery for inguinal hernia repair enables observation of the inguinal region and management of rare cases, such as retroperitoneal liposarcoma. We present a rare case of retroperitoneal liposarcoma with an inguinal swelling, which was diagnosed after laparoscopic surgery for inguinal hernia. It is necessary to consider a possibility of enlargement of retroperitoneal liposarcoma and perform CT or MRI of the abdomen and pelvis in cases of irreducible inguinal swelling of retroperitoneal fatty tissue.

## Declaration of Competing Interest

No conflicts of interest.

## Sources of funding

We have no sponsor.

## Ethical approval

In National Hospital Organization Kumamoto Medical Center, we had forgiven from our ethical approval. The number of our judgement of ethics committee and the state is 907.

## Consent

Written informed consent was obtained from the patient for publication of this case report and accompanying images. A copy of the written consent is available for review by the Editor-in-Chief of this journal on request.

## Author contribution

All authors read and approved the manuscript. TM drafted the manuscript. TM, AO, KM, and NM performed the surgery and critically revised the manuscript. AM, SY, KK, MI, TM, TK, and HB performed the investigation and critically revised the manuscript.

## Registration of research studies

NA.

## Guarantor

Kosuke Mima.

## Availability of data and materials

Data sharing is not applicable to this article as no datasets were generated or analyzed during the current study.

## Provenance and peer review

Not commissioned, externally peer-reviewed.
